# BoaγPLI: Structural and functional characterization of the gamma phospholipase A2 plasma inhibitor from the non-venomous Brazilian snake *Boa constrictor*

**DOI:** 10.1371/journal.pone.0229657

**Published:** 2020-02-27

**Authors:** Caroline Fabri Bittencourt Rodrigues, Caroline Serino-Silva, Karen de Morais-Zani, Victor Koiti Kavazoi, Marcelo Pires Nogueira Carvalho, Kathleen Fernandes Grego, Tassia Chiarelli, Alexandre Keiji Tashima, Marcos Hikari Toyama, Anita Mitico Tanaka-Azevedo

**Affiliations:** 1 Interunidades em Biotecnologia, Universidade de São Paulo—Instituto de Pesquisas Tecnológicas—Instituto Butantan, São Paulo, São Paulo, Brazil; 2 Laboratório de Herpetologia, Instituto Butantan, São Paulo, São Paulo, Brazil; 3 Universidade Federal de Minas Gerais, Belo Horizonte, Minas Gerais, Brazil; 4 Departamento de Bioquímica, Universidade Federal de São Paulo, São Paulo, São Paulo, Brazil; 5 Instituto de Biociências do Litoral Paulista, Universidade Estadual Paulista, São Vicente, São Paulo, Brazil; Weizmann Institute of Science, ISRAEL

## Abstract

Plasma in several organisms has components that promote resistance to envenomation by inhibiting specific proteins from snake venoms, such as phospholipases A_2_ (PLA_2_s). The major hypothesis for inhibitor’s presence would be the protection against self-envenomation in venomous snakes, but the occurrence of inhibitors in non-venomous snakes and other animals has opened new perspectives for this molecule. Thus, this study showed for the first time the structural and functional characterization of the PLA_2_ inhibitor from the *Boa constrictor* serum (BoaγPLI), a non-venomous snake that dwells extensively the Brazilian territory. Therefore, the inhibitor was isolated from *B*. *constrictor* serum, with 0.63% of recovery. SDS-PAGE showed a band at ~25 kDa under reducing conditions and ~20 kDa under non-reducing conditions. Chromatographic analyses showed the presence of oligomers formed by BoaγPLI. Primary structure of BoaγPLI suggested an estimated molecular mass of 22 kDa. When BoaγPLI was incubated with Asp-49 and Lys-49 PLA_2_ there was no severe change in its dichroism spectrum, suggesting a non-covalent interaction. The enzymatic assay showed a dose-dependent inhibition, up to 48.2%, when BoaγPLI was incubated with Asp-49 PLA_2_, since Lys-49 PLA_2_ has a lack of enzymatic activity. The edematogenic and myotoxic effects of PLA_2_s were also inhibited by BoaγPLI. In summary, the present work provides new insights into inhibitors from non-venomous snakes, which possess PLIs in their plasma, although the contact with venom is unlikely.

## 1. Introduction

Snake envenomation, reclassified as a neglected tropical disease by the World Health Organization (WHO), can have serious pathophysiological consequences [1–3]. The pharmacological actions of envenomation are related to the toxins’ actions present in the venom, which consist mainly of proteins, whose activities can promote homeostatic, neuromotor, inflammatory and blood clotting disorders. Among the enzymatic proteins commonly found in the venoms are metalloproteases (SVMP), serine proteases (SVSP), phospholipases A_2_ (PLA_2_) and L-amino acid oxidases (LAAO) [4–6].

The PLA_2_s are a group of low molecular mass enzymes (~ 13 to 15 kDa), which are related to calcium-dependent cleavage at the sn-2 position of phospholipids, releasing lysophospholipids and arachidonic acid, the precursor of the inflammatory cascade [7]. PLA_2_s can be divided into several groups, being that those present in the Viperidae family snakes belong to group II and can be separated into two subgroups: Asp49-PLA_2_ and Lys49-PLA_2_. The variant Asp49-PLA_2_ has a high enzymatic activity. When there is a substitution of the amino acid residue at position 49, the most common being Lys-49 substitution, there is a loss in the ability of calcium binding, resulting in a severe reduction of its enzymatic activity [8,9].

Nevertheless, PLA_2_s pharmacological actions are not only related to their enzymatic activity, being responsible for myotoxicity, neurotoxicity and inflammatory disorders in snake bite envenomation. This protein is also responsible for local tissue damage, lethality and irreversible effects, such as muscle damage and loss of limbs, leading to individual incapacitation [10–12]. Furthermore, they also have anticoagulant, cardiotoxic, and platelet aggregation-inducing / inhibitory activity [8,13]. Several molecules have an inhibitory capacity against PLA_2_s activity, some of which were identified by transcriptome of liver or isolated from snake plasma [14–17]. *In silico* techniques were also used to search for potential inhibitors [18].

The major hypothesis for the presence of PLA_2_ inhibitors (PLIs) in venomous snakes is the protection against self-envenomation. However, such theory does not support their presence in non-venomous snakes [19–22], whose occurrence suggests that its physiological role is not restricted to protection against self-envenomation, but has a role not yet completely understood [23].

PLIs can be homo or hetero-oligomeric and are usually glycoproteins, but the carbohydrate is not essential for its inhibitory activity [14]. Due to their structural differences, such inhibitors can be classified into three groups: αPLI, βPLI, and γPLI, whose domains are related to the interaction between the inhibitor and PLA_2_ [16].

Regarding the γPLIs, they are characterized by two structural units of highly conserved cysteine repeats, known as three finger motifs [24]. Another important feature of γPLIs is the highly conserved proline-rich region, that plays an important structural role, ensuring the integrity and conformation of protein interaction sites [25].

The γPLI from *Boa constrictor* was already identified by transcriptomic analysis [26], but its functional characterization has not been reported yet. Given the background, the biotechnological potential of these inhibitors may provide therapeutic molecular models with antiophidic activity to complement conventional serum therapy against these multifunctional enzymes, as well as its anti-inflammatory potential, since there is a structural and catalytic similarity between venom and human PLA_2_s, besides contributing to the elucidation of the PLA_2_-PLI interaction mechanism. In this context, we isolated a γPLI from *Boa constrictor* plasma, named as BoaγPLI, and characterized it structurally (primary and secondary structure, and its oligomerization) and functionally by enzymatic and pharmacological effects such as edema and myotoxicity inhibition of Asp-49 and Lys-49 PLA_2_ activity.

## 2. Material and methods

### 2.1. Ethical committee

All animal experiments were approved by the Ethical Committee of Instituto Butantan (protocol number 6916110917) and experiments were in accordance with the Brazilian laws for the use of experimental animals and with the ethical principles adopted by the Brazilian College of Animal Experimentation (COBEA).

### 2.2. Snake venom

Snake venoms from *Crotalus durissus terrificus* (three captivite animals) and *Bothrops jararacussu* (three captivite animals) were provided by the Laboratory of Herpetology at Instituto Butantan. Venoms were obtained by manual extraction, centrifugated at 1700 g for 15 minutes, lyophilized and stored at -20°C.

### 2.3. Serum

The blood of five *Boa constrictor* from captivity was collected by puncture of the paravertebral vein using plastic syringes and pooled. The volume of blood collected corresponded to 1% of snake total weight. Blood was maintained for 18 hours at 4°C for coagulation, prior to centrifugation (1200 g, 15 minutes) and stored at -20°C.

### 2.4. Purification of Asp-49 PLA_2_ from *Crotalus durissus terrificus* and Lys-49 PLA_2_ from *Bothrops jararacussu*

Purification of an Asp-49 PLA_2_ from *C*. *d*. *terrificus* venom was performed according to Oliveira et al., (2002) [27]. The venom was fractionated on a gel-filtration chromatography, using a Superdex 75 column (GE Healthcare 10/300) and 50 mM Tris 100 mM NaCl pH 7.4 buffer, for 250 minutes at 1 mL/min. Subsequently, for some tests, crotoxin A (crotapotin) was dissociated from crotoxin B (PLA_2_) by a reverse phase C5 column chromatography (Supelco C5 column, 0.10 cm × 25 cm) [28]. The chromatographic column was pre-equilibrated with solution A (0.1% TFA). Elution of PLA_2_ was performed with a continuous linear gradient of solution B (66% acetonitrile in 0.1% TFA) and monitoring the chromatographic profile at 280 nm (detector UV-2077, Jasco, Japan). The samples were then lyophilized.

The purification of a Lys-49 PLA_2_ from *Bothrops jararacussu* (Lys-49 PLA_2_) was performed following Soares et al., (1998) [29]. The venom (100 mg) was fractionated on an ion exchange CM column (GE Healthcare, 5 mL), with a linear gradient (0–100%) of 0.05 M ammonium bicarbonate buffer to 1 M ammonium bicarbonate buffer pH 7.9, monitored by 215 nm. The fraction containing the purified PLA_2_ was lyophilized.

### 2.5. Purification of PLI

The purification of PLI from *Boa constrictor* serum was performed in two chromatographic steps according to Serino-Silva et al., (2018) [30], with some modifications. In the first step, the *Boa constrictor* serum (5 mL) was diluted in 5 mL of 25 mM Tris buffer pH 7.5 (buffer A) and applied to an anion exchange column (HiTrap DEAE FF 5 mL, GE Healthcare), previously equilibrated with 95% buffer A (25 mM Tris, pH 7.5) and 5% buffer B (25 mM Tris, 1 M NaCl pH 7.5). Elution was performed maintaining 10% buffer B (100 mM NaCl) and then with a gradient of buffer B up to 50% (500 mM NaCl). The run was maintained at a flow rate of 1 mL/min, monitored at 280 nm, and the samples were fractionated every 5 mL (Akta purifier, GE Healthcare). After the chromatography, the protein fractions were pooled. Pool desalination was done by dialysis in PBS (140 mM NaCl, 2.6 mM KCl, 10 mM Na_2_HPO_4_, 1.7 mM KH_2_PO_4_, pH 7.4) for 24 hours at 4°C in a 10000 MWCO membrane. Then, the D2 fraction from DEAE chromatography was applied to an affinity column, previously prepared by the coupling of crotoxin into a CNBr-activated Sepharose matrix (GE Healthcare) and equilibrated with PBS. The non-adsorbed material was removed by extensive washing with PBS. Finally, the proteins adsorbed to the resin were eluted with 0.1 M glycine pH 2.7, fractionated in microtubes, and the pH of the fractions was neutralized by the addition of 1 M Tris buffer pH 8.8 (9:1 v/v). The elution was manually measured in a spectrophotometer (Spectramax, Molecular Device) at 280 nm.

### 2.6. SDS-containing polyacrylamide gel electrophoresis (SDS-PAGE)

PLI samples were subjected to 12% SDS-PAGE, according to Laemmli, (1970) [31] under reducing by β- mercaptoethanol or non-reducing conditions. By lane, 20 μg of protein was applied. The molecular marker used was Dual Color Precision Plus, BioRad. The gels were stained using Coomassie Blue R350 (GE Healthcare).

### 2.7. Mass spectrometry

Protein bands were excised from SDS-PAGE (under reducing conditions) which were dehydrated with acetonitrile addition and subjected to reduction with 5 mM dithiothreitol for 30 min at 60°C, alkylation with 15 mM iodoacetamide for 30 min under light protection at room temperature and overnight in-gel digestion with sequencing grade trypsin (Sigma), in 50 mM ammonium bicarbonate at 37°C. Digested peptides were analyzed by a Synapt G2 mass spectrometer coupled to a nanoAcquity UPLC system (Waters). Samples were injected into a trap column (C18 nanoAcquity trap Symmetry column 180 μm x 20 mm, Waters) with 0.1% (v / v) formic acid. Peptides were eluted on a capillary analytical column (C18 nanoAcquity BEH 75 μm x 150 mm, 1.7 μm column) using a gradient of 93% A (0.1% formic acid) and 7% B (99.9% ACN 0.1% formic acid) to 35% B over 30 min in a flow of 275 nl / min. Data were acquired in MS^E^ mode [32,33] in duplicate. Protein identification, PTM and homology searches were performed in PEAKS Studio 7.5 software (Bioinformatics Solutions Inc.) by MS/MS search against the Serpentes databases obtained from Uniprot (2844 sequences, downloaded in October 25^th^, 2018). Analyses were carried out with precursor mass tolerance of 10 ppm, fragment mass tolerance of 0.025 Da and peptide cleavage by trypsin. Carbamidomethylation of the cysteines was considered as a fixed modification and the oxidation of methionines, N-terminal acetylation, and deamidation of asparagines and glutamines as variable modifications. Assignments for peptides and proteins were accepted at a false discovery rate < 1%. As the inhibitor was indentified with γPLIs, it was named as BoaγPLI.

### 2.8. Size-exclusion chromatography protein analysis (SEC)

BoaγPLI (20 μg) were subjected to size-exclusion chromatography (BioSep SEC-s2000, Phenomenex) using 0.05 M Tris HCl, 0.05 M NaCl, pH 8, at a flow rate of 1 mL/min, and with monitoring at 280 nm (MD-2018, Jasco). For comparison of molecular mass, Gel Filtration Standard (BioRad) was used.

### 2.9. Circular dichroism

The Asp-49 and Lys-49 PLA_2_ (30 μg), BoaγPLI (30 μg) or the incubated mixture (PLA_2_-PLI, 1:1) (w/w) was subjected to circular dichroism assay (J815 spectropolarimeter, Jasco). Proteins diluted in 0.002 M Tris 0.015 M NaCl 0.1 mM CaCl_2,_ pH 8 were run at wavelengths of 190 to 260 nm (1 nm/s) under 8 convolutions. All analyses were subtracted from the buffer used. The data were exported by Spectra manager and the relative percentages of secondary protein structures were determined by Circular Dichroism analysis using Neural Networks (CDNN) software.

### 2.10. Inhibition of the PLA_2_ enzymatic activity

The PLA_2_ activity was performed according to Holzer and Mackessy, (1996) [34]. The enzyme (*C*.*d*. *terrificus* Asp-49 PLA_2,_ 1 mg/mL) and the inhibitor (with a range of concentrations of 2 mg/mL, 1.5 mg/mL, 1 mg/mL and 0.5 mg/mL) were incubated for 10 minutes. Then, 20 μL of each solution were applied to the plate. The chromogenic substrate 4-nitro-3-octanoyloxy benzoic acid (NOB) (Enzo Life Sciences) (solubilized in 3 mM acetonitrile PA) was used (20 μL). Afterwards, the buffer 0.01 M Tris-HCl, 0.10 M NaCl and 0.01 M CaCl_2,_ pH 8 was added to the samples (200 μL). The controls were composed by 20 μL of saline 0.85%, 20 μL of inhibitor (2 mg/mL) or 20 μL of PLA_2_ (1 mg/mL)_._ The activity was analyzed by spectrophotometer Spectramax (Molecular Devices) at 425 nm, and the readings occurred over 90 minutes, with 5-minute intervals between readings. The absorbances of the last reading were transformed into the PLA_2_ specific activity as nmol/mg/min per unit. Then, the percentage of inhibition was determined. The experiment was done in triplicate.

### 2.11. Paw edema inhibition

The paw edema was induced by a subplantar injection in male Swiss mice (18 to 21 g; n = 5) of 10 μg of Asp-49 or Lys-49 PLA_2_, previously incubated for 30 min with 20 μg of BoaγPLI with a final volume injection of 20 μL. The contralateral paw was injected with the same volume of sterile 0.85% NaCl solution. Control groups were inoculated with 20 μg of BoaγPLI, 0.85% NaCl, or 10 μg of Asp- 49 or Lys- 49. Paw thickness was measured using a caliper reading to 0.01 mm at 0, 0.5, 1, 2, 4, 6 and 24 h after injection. Results were expressed as the difference in thickness of both paws and represented as the percentage increase in paw thickness.

### 2.12. Myotoxicity inhibition

The myotoxicity inhibition by BoaγPLI was determined according to Belchor et al. (2017) [35], by the injection of 40 μL of Asp-49 or Lys-49 PLA_2_ (10 μg) incubated for 30 min with BoaγPLI (20 μg) on the right *gastrocnemius* muscle (18 to 21 g male Swiss mice, n = 5). Control groups received 20 μg of Asp-49 or Lys-49 PLA_2_ or 20 μg of BoaγPLI or 40 μL of 0.85% saline. Mice blood samples were collected from the tip of the tail [36] into tubes containing citrate as anticoagulant, centrifuged at 1200 g for 15 minutes and the plasma was separated. The amount of Creatine Kinase (CK) present in the samples was estimated with a commercial CK kit (Sigma), according to the manufacture’s instructions.

### 2.13. Statistical analyses

The data were expressed as mean ± standard deviation (SD). The enzymatic and myotoxic assays were analyzed with one-way ANOVA, with Tukey as *a posteriori* test, while the edematogenic test was analyzed with a two-way ANOVA, with Tukey as *a posteriori* test. The inhibition of the edema activity was specified by the area under the curve analysis. Values of p<0.05 was considered significant.

## 3. Results and discussion

This work shows for the first time the structural and functional characterization of the BoaγPLI, a PLI from *Boa constrictor*, a non-venomous Brazilian snake. Contrasting with the number of PLIs isolated from venomous snakes [14,16], only a small number of this molecule were isolated from non-venomous snakes [19,22], suggesting that the PLI is not restricted to protection against self-envenomation, but it may be involved in other unknown physiological mechanisms that has yet to be determined [23].

**Fig 1:**
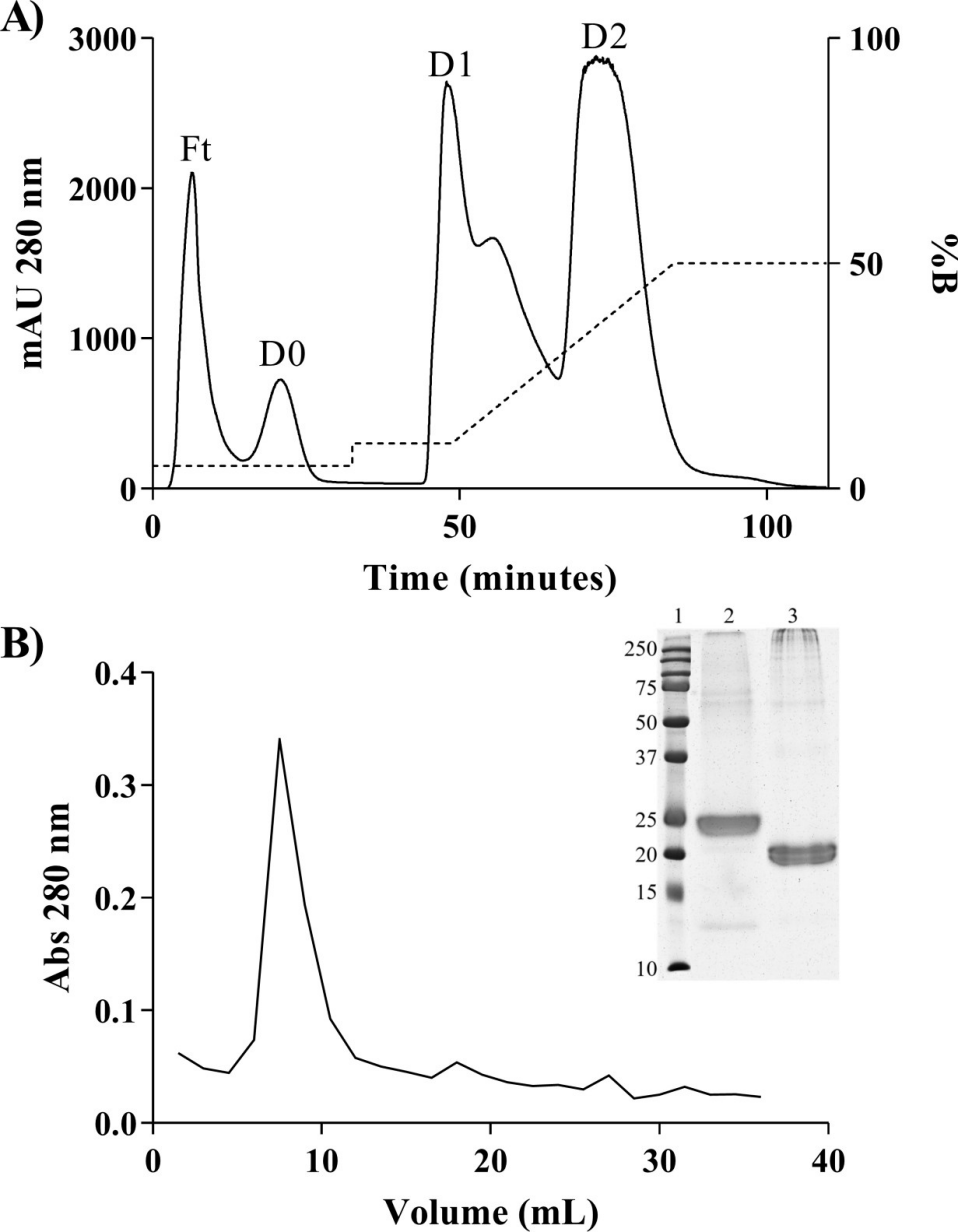
The purification process of the BoaγPLI. A) Fractionation of *B*. *constrictor* serum. HiTrap DEAE column ion exchange chromatography (5 mL), flow 1 mL / min. Five ml of serum diluted in buffer A were applied to the chromatography. Three resulting fractions were evidenced: D0, D1, and D2. B) Elution of D2 fraction retained on CNBr-activated Sepharose resin-coupled crotoxin affinity chromatography (GE Healthcare). Elution was performed with 0.1 M glycine, pH 2.7, pH neutralized with the addition of 1 M Tris, pH 8.8. Elution was manually dosed at 280 nm. Insert: SDS-Page 12% of the BoaγPLI in reducing (2) and non-reducing conditions (3). 1) Molecular mass marker Dual Color Precision Plus, Biorad.

In this study, was performed using two chromatographic steps (Fig 1) and the percentage of recovery was 0.63% (Table 1). a reasonable value considering that the inhibitor is a minor protein of the serum, especially in a non-venomous species, whose contact with venom is unlikely. However, the value was superior to the percentage of recovery from *Phyton reticulatus*’ PLI, which was 0.25% [37], although the distinct methodology applied could have affected the recovery.

**Table 1:** Purification table of the *Boa constrictor* PLI.

	Total mg*	% of recovery
Initial	125.15	-
D2	57.81	46.19%
Eluted (BoaγPLI)	0.80	0.63%

*mensured by A_280_ nm.

In comparison, the purification of the γPLI from *Bothrops jararaca* (γBjPLI) presented 1% of recovery, using the same methodology applied in this case [30], while under different approaches the γPLI from *Crotalus durissus collilineatus* (γCdcPLI) presented 2.69% [38]. The higher recovery of PLIs from venomous snakes may be related to the constant contact with the venom, which may stimulate the presence of inhibitors in the plasma [39].

In fact, a difference in the PLIs recovery percentage between two non-venomous snakes of the same genus (*Elaphe quadrivirgata* and *Elaphe climacophora*) was observed. This behavior can be related to its dietary habits, since *E*. *quadrivirgata* may be ophiophagus, which would result in an indirect contact with venom. Consequently, this animal can have its inhibitors positively regulated [19]. Nevertheless, contact with venom may not be the only mechanism related to the presence of PLIs in snake’s plasma.

The SDS-PAGE of BoaγPLI after elution of affinity chromatography, showed a band at 25 kDa, under reducing conditions, and 20 kDa, under non-reducing conditions. Actually, data in the literature suggest a monomerization when inhibitors had contact with PLA_2_ [40]. Thus, since affinity chromatography has crotoxin coupled in the resin, the contact of the D2 fraction with it probably monomerizes the inhibitor, explaining the result found in the electrophoresis (Fig 1, insert). BoaγPLI had an anomalous migration under reducing conditions, in which it has a higher molecular mass than under non-reducing conditions. Molecular interactions of PLI, such as non-covalent bonds and disulfide bonds, might facilitate migration under non-reducing conditions. When the molecule is linearized, in turn, a slower migration occurs [40]. In addition, the same phenomenon was observed with γBjPLI [30].

**Fig 2:**
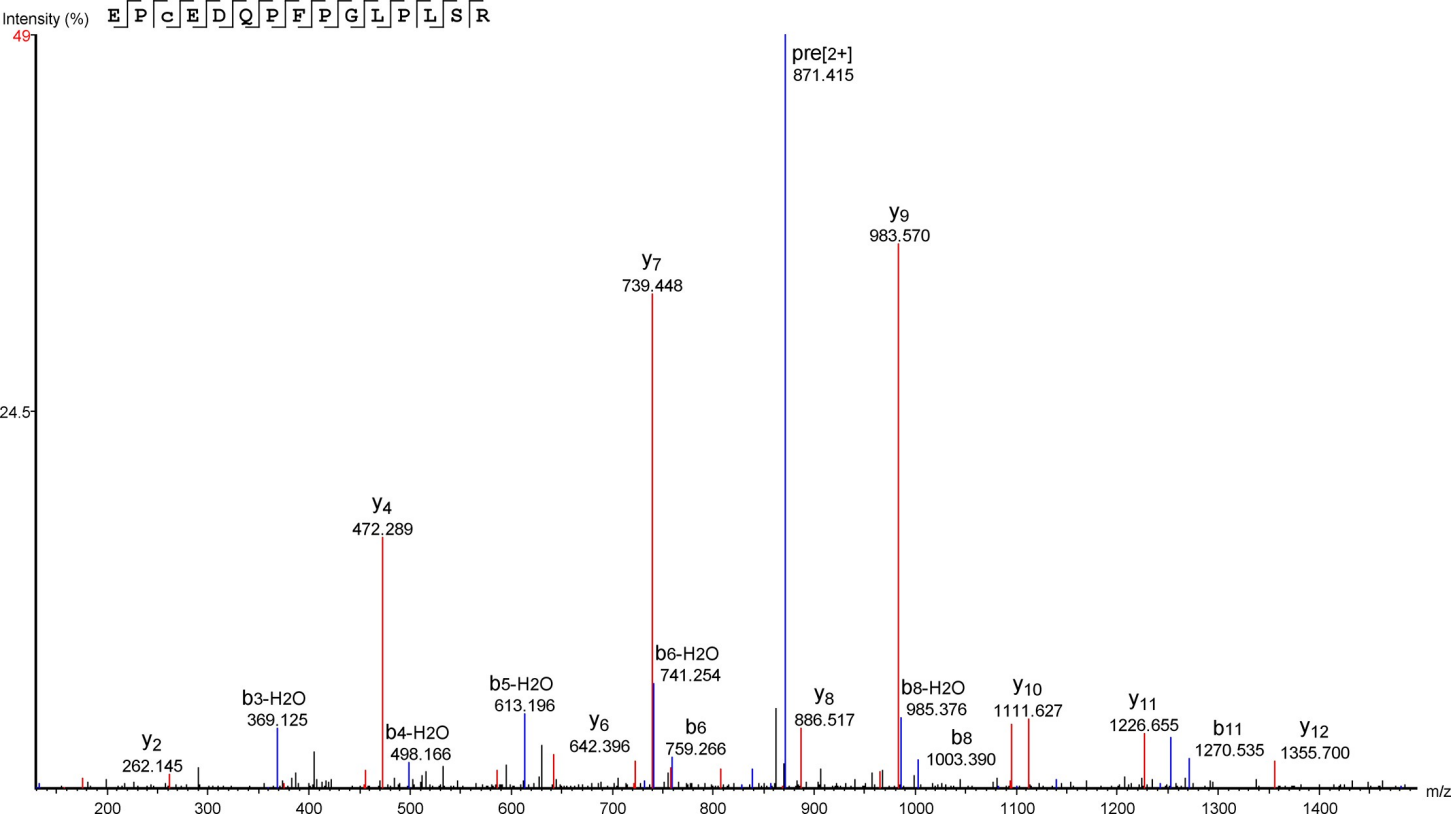
MS/MS spectrum of the BoaγPLI peptide. Annotated MS/MS spectrum of the tryptic peptide EPCEDQPFPGLPLSR from the BoaγPLI. The peptide was detected at m/z 871.42^+2^ and identified by database search in PEAKS Studio 7.5.

The partial amino acid sequence of BoaγPLI has been identified (best MS/MS spectrum match shown in Fig 2), with 58% and 30% of coverage with the γPLI subunit from *Lachesis muta* (P60591), 30% with PLI from *C*. *d*. *terrificus* (Q90358), 32% coverage with *Glodyus brevicaudus siniticus* (P82143) and 17% with *Protobothrops flavoridis* (O57690) and *Elaphe quadrivirgata* (Q9PWI4), the last being a non-venomous snake (Table 2), reinforcing the classification of the BoaγPLI as a γPLI.

**Table 2:** Coverage of the peptides from BoaγPLI. Identification of the proteins with a higher coverage from the peptides obtained from SDS-PAGE band excised and analyzed by mass spectrometry.

Access number	Coverage (%)	Peptides	Mass (Da)	Description
P60592	58	30	22207	Phospholipase A_2_ inhibitor LNF2 OS = *Lachesis muta muta*
P60591	30	12	22235	Phospholipase A_2_ inhibitor LNF1 OS = *Lachesis muta muta*
Q90358	30	12	22267	Phospholipase A_2_ inhibitor CNF OS = *C*. *durissus terrificus*
P82143	32	11	22232	Phospholipase A_2_ inhibitor subunit γ B OS = *G*. *b*.*siniticus*
O57690	17	9	22395	Phospholipase A_2_ inhibitor 1 OS = *Protobothrops flavoviridis*
Q9PWI4	17	7	22547	Phospholipase A_2_ inhibitor subunit γ A OS = *E*. *quadrivirgata*

When the inhibitor was identified by mass spectrometry, the peptides identified presented similarities with 6 previously described PLIs and it is possible to observe that the identified sequence of BoaγPLI is well conserved compared to other PLIs (Table 2). In addition, the γPLI sequence of *Lachesis muta* was identified through its transcript [41], having its signal peptide of 19 amino acids, which is absent in a purified plasma protein. Disregarding the signal peptide, the coverage increases to 64%.

The oligopeptide ^104^QPFPGLPLSRPNGYY^118^ was suggested as a site of interaction between γPLIs and PLA_2_s in *Bothrops sp* [42] and this amino acid sequence was partially identified in BoaγPLI (part of this sequence shown in Fig 2), which reinforces the conserved primary structure of these inhibitors [30,40]. Some authors also suggest that there may be three phosphorylation sites, at position ^21^S, ^22^S and ^111^T, and that these sites may be involved in other physiological roles, beyond the inactivation with PLA_2_, which would reinforce the hypothesis about their presence in plasma of non-venomous snakes. So far, no other role has been assigned to PLIs, and these phosphorylation sites were not found in BoaγPLI partial sequence [30,40,42].

**Fig 3:**
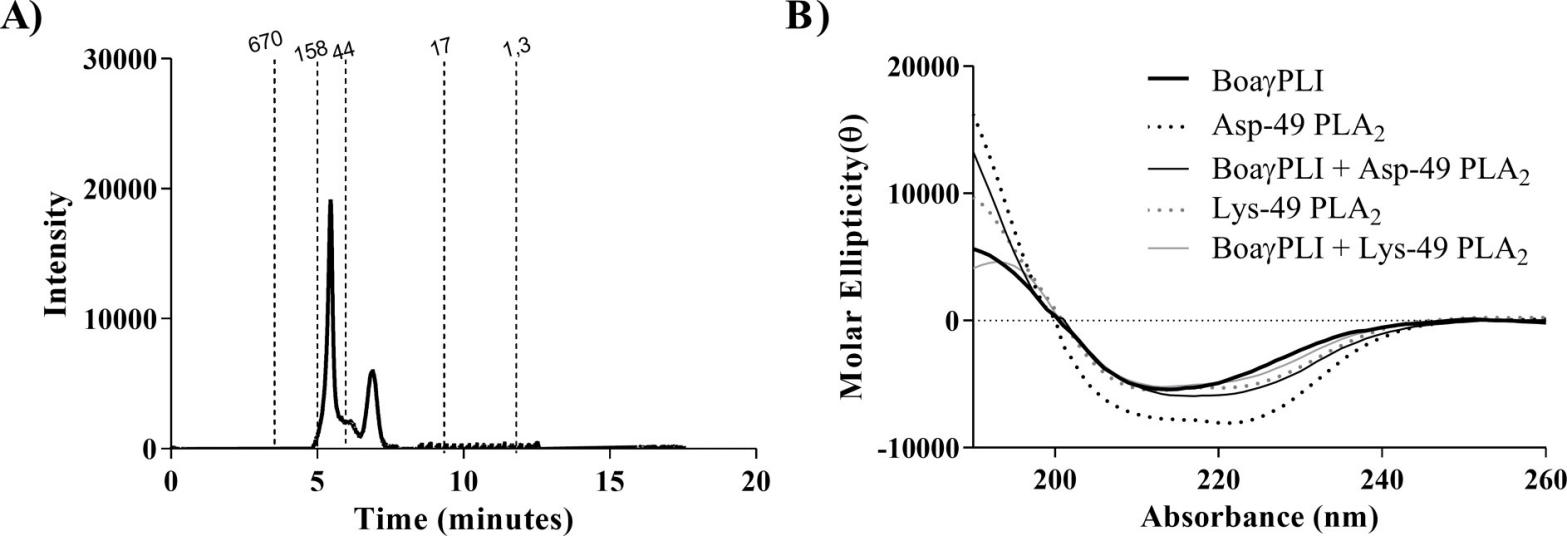
Structural analysis of the BoaγPLI. A) Chromatographic profile on BioSep SEC S-2000 size-exclusion chromatography of BoaγPLI (20 *μ*g), at a flow of 1 mL / min, monitored by 280 nm. B) Spectra of Asp-49 and Lys-49 PLA_2_s isolated, BoaγPLI isolated and PLA_2_s + BoaγPLI incubated (30 μg) obtained by circular dichroism. The data were expressed in molar ellipticity.

The size-exclusion chromatography showed two peaks corresponding to oligomer and monomer, respectively (Fig 3A). This result is corroborated by the γPLI homologue from *Python sabae*, that also showed oligomers and monomers in its spectrum [43]. On the other hand, γBjussuMIP [40] showed only the oligomer, as well as the purified γPLI of *Macropisthodon rudis* [20,40]. Our group has found only the monomeric form of γBjPLI. Moreover, the interaction of γBjPLI with Asp-49 PLA_2_ was also evidenced in this work [30].

BoaγPLI secondary structure was investigated by circular dichroism (Fig 3B), presenting 21.4% of alpha-helices, 39.4% of beta-sheets and 38.7% of random coils. Besides that, incubation of BoaγPLI with Asp-49 and Lys-49 PLA_2_s does not significantly alter the spectrum shown in circular dichroism, which was also evidenced in γCdcPLI and γBjussuMIP [38,40]. This observation suggests a weak interaction of BoaγPLI with PLA_2_, most likely non-covalently. The PLI of *Macropisthodon rudis* also showed non-covalent binding characteristics evidenced by SDS-PAGE when incubated with PLA_2_ under non-reducing conditions [20].

**Fig 4:**
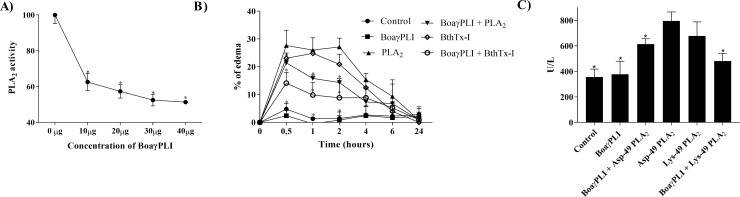
Inhibitory potential of the BoaγPLI. A) Enzymatic activity of Asp-49 PLA_2_ incubated with different concentrations (0 μg, 10 μg; 20 μg and 40 μg) of BoaγPLI with NOB as a substrate. * represents statistical differences when compared to 0 μg of inhibitor. B) Edematogenic activity of Asp-49 and Lys-49 PLA_2_ isolated and when previously incubated for 30 min with 20 μg of BoaγPLI, injected into the right sub plantar region of mice paw (Swiss). C) Myotoxic activity of Asp-49 and Lys-49 PLA_2_ isolated and when previously incubated for 30 min with 20 μg of BoaγPLI, injected in the *gastrocnemius* muscle of mice (Swiss). * represents statistical differences when compared to PLA_2_s.

Once the possible interaction between BoaγPLI and PLA_2_s was evidenced, an inhibition assay was performed (Fig 4A), resulting in a dose-dependent inhibition, reaching 48.7% when 40 μg of inhibitor were used, values similar to found for γBjPLI [30]. In contrast, a γPLI inhibitor from *Python sabae* showed a low enzymatic activity inhibition of PLA_2_ from bee venom. In turn, PIP, the γPLI isolated from *Python reticulatus*, showed a high inhibition at 1:1 molar ratio, reaching 90%, measured by egg yolk acidimetric method, with PLA_2_ of *D*. *r*. *russelli* [37].

The inhibitory potential of BoaγPLI was also verified considering edematogenic and myotoxic effects of Asp-49 and Lys-49 PLA_2_s. Wherein, when incubated with the inhibitor, edema was significantly lower compared to isolated PLA_2_s. In order to highlight the differences between the curves of treatments, the area under the curve was estimated. As expected, edema profile induced by isolated PLA_2_s resulted in a higher area under the curve when compared to the groups that received PLA_2_s pre-incubated with BoaγPLI (Control: 58.66; BoaγPLI: 43.51; Asp-49 PLA_2_: 206.0; BoaγPLI + Asp-49 PLA_2_: 134.8; Lys-49 PLA_2_: 126.5; BoaγPLI + Lys-49 PLA_2_: 105.7). (Fig 4B). Creatine kinase levels, which reflects myotoxicity, were also reduced when PLA_2_s were incubated with the inhibitor (Fig 4C). Other γPLIs from venomous and non-venomous snakes also demonstrated the inhibitory potential in the pharmacological activities caused by PLA_2_ [30,38,40]. In addition, our data showed no statistical differences of the inhibition potential of BoaγPLI against Asp-49 and Lys-49 PLA_2_s, unlike γBjussuMIP, which possess higher affinity for Asp-49 PLA_2_ [40]_._ An interesting point of the study is that, even being a non-venomous snake, the functional and structural characteristics of BoaγPLI were well conserved when compared with γPLIs from venomous snakes.

## 4. Concluding remarks

In summary, the study showed the purification of a γPLI of the non-venomous snake *Boa constrictor* (BoaγPLI), which has a molecular mass of approximately 22 kDa, oligomerization capacity, and a primary structure similar to the PLI of *Lachesis muta*. The direct interaction of BoaγPLI with Asp-49 PLA_2_ of *C*. *d*. *terrificu*s and Lys-49 PLA_2_ from *Bothrops jararacussu* was evidenced by circular dichroism, and the molecule displayed inhibition upon enzymatic, edematogenic and myotoxic activities of PLA_2_s.

An interesting point of this study is that our data add another piece of evidence pointing the wide distribution of these inhibitors, that appears in venomous and non-venomous snakes, and, beyond that, their structure and inhibitory activity seems to be well conserved between them. That evidence amplifies the primary hypothesis for the PLI presence, that was the protection against the self-envenomation. Therefore, the inhibitor is not restricted to such function.

In addition, since γPLIs inhibit PLA_2_s from IIA group, which includes proinflammatory PLA_2_s from mammalian and from viperids, the isolation and characterization of distinct γPLIs may contribute to the enrichment of information for the bioprospection that can be useful, not only in antivenom therapy, but also in other inflammatory processes triggered by human PLA_2_s, whereas their catalytic site is well conserved [8,42]. In this context, the present study may contribute to the elucidation of the presence of PLA_2_ inhibitors in non-venomous snakes and to provide new perspectives for PLA_2_ inhibitors from snake plasma by characterizing and comparing the similarities and differences between PLI from venomous and non-venomous snakes.
